# Awake burr hole craniotomy for chronic subdural hematoma: a phase 2 randomized controlled trial

**DOI:** 10.1186/s13054-026-05913-1

**Published:** 2026-03-09

**Authors:** Robert Mertens, Clara F. Weber, Lukas Depperich, Philipp Spindler, Claudius Jelgersma, Kiarash Ferdowssian, Anton Früh, Peter Truckenmüller, Ahmad Almahozi, Erin D. Sprünken, Theresa Keller, Anika Müller, Alawi Lütz, Claudia Spies, Friederike Töpper, Sascha Treskatsch, Nils Hecht, Peter Vajkoczy, Lars Wessels

**Affiliations:** 1 Department of Neurosurgery, Charité – Universitätsmedizin Berlin, Corporate Member of Freie Universität Berlin and Humboldt-Universität zu Berlin, Charitéplatz 1, 10117 Berlin, Germany; 2 Institute of Biometry and Clinical Epidemiology, Charité – Universitätsmedizin Berlin, Corporate Member of Freie Universität Berlin and Humboldt-Universität zu Berlin, Berlin, Germany; 3 Department of Anesthesiology and Intensive Care Medicine (CCM/CVK), Charité – Universitätsmedizin Berlin, Corporate Member of Freie Universität Berlin and Humboldt-Universität zu Berlin, Berlin, Germany; 4 Department of Anesthesiology and Intensive Care Medicine (CBF), Charité – Universitätsmedizin Berlin, Corporate Member of Freie Universität Berlin and Humboldt-Universität zu Berlin, Berlin, Germany

**Keywords:** Chronic subdural hematoma, Local anesthesia, General anesthesia, Postoperative delirium

## Abstract

**Introduction:**

Chronic subdural hematoma (cSDH) is a common condition in older adults that often requires neurosurgical evacuation. Postoperative delirium is a frequent and clinically relevant postoperative complication in this population. This study investigated whether burr hole craniotomy for cSDH performed under local anesthesia (LA) reduces the risk of postoperative delirium and complications compared to general anesthesia (GA).

**Methods:**

The ABC-SDH trial was a single-center, open-label, phase 2, prospective randomized clinical trial conducted at a tertiary academic medical center between October 2023 and November 2024. Fifty consecutive patients with a confirmed diagnosis of cSDH underwent burr hole craniotomy performed under either LA or GA. Primary outcomes were postoperative delirium (Confusion Assessment Method, CAM) and complications rates until discharge, and the recruitment rate. Secondary outcomes included procedural times and clinical outcome measures at discharge and at 30-day follow-up.

**Results:**

LA was associated with a significantly lower rate of postoperative delirium (4% vs. 32%, OR 0.09; 95%CI, 0.01–0.79; *p* = 0.03; NNT = 4). Complications occurred in 8% (LA) versus 32% (GA) (OR 0.18, 95% CI 0.03–0.96; *p* = 0.08). LA was also associated with significantly reduced procedural times (139.3 ± 53.0 vs. 196.0 ± 52.1 min; *p* = 0.002). The severity of complications and functional outcomes were comparable between groups.

**Conclusion:**

LA was associated with significantly lower rates of postoperative delirium and a shorter periprocedural duration compared to GA in patients undergoing cSDH evacuation. As the first prospective trial to systematically assess the incidence of delirium following burr hole craniotomy for cSDH, the ABC-SDH trial highlights the feasibility and safety of LA in a delirium-prone population.

**Supplementary Information:**

The online version contains supplementary material available at 10.1186/s13054-026-05913-1.

## Introduction 

Chronic subdural hematoma (cSDH) is a prevalent condition among older adults. By 2030, it is projected to become the most common cranial neurosurgical condition [1], emphasizing the need for evidence-based, age-adapted management strategies. Burr hole craniotomy is the standard surgical approach for the evacuation of cSDH [2]. While this procedure is considered routine and safe, the elderly population most at risk for cSDH is also particularly vulnerable to postoperative complications associated with general anesthesia (GA). One of the most common and serious complications is postoperative delirium, characterized by an acute change in attention and awareness, accompanied by a disturbance in cognition, and not better explained by a pre-existing neurocognitive disorder or reduced level of arousal [3]. Surgery and GA are major precipitating factors for delirium, which is associated with impaired recovery, prolonged hospitalization, and increased mortality, contributing to substantial socioeconomic burden [4–6]. Given the susceptibility of older patients to GA-related complications, burr hole craniotomy under local anesthesia (LA) has been suggested as a safe and viable alternative, with lower complication rates, reduced hospital stay, and comparable recurrence and mortality rates [7]. Nevertheless, available evidence remains scarce and is primarily based on retrospective studies. In particular, there is no evidence that LA affects the incidence of postoperative delirium in cSDH. As a result, burr hole craniotomy under LA has not yet been established as standard of care in clinical practice [7, 8]. To address this knowledge gap, the Awake Burr Hole Craniotomy for Chronic Subdural Hematoma (ABC-SDH) trial was conducted as a prospective, randomized safety and feasibility trial comparing postoperative delirium rate, complications, and clinical outcomes in patients undergoing burr hole craniotomy for cSDH under GA compared with LA.

## Methods

The ABC-SDH trial was a parallel-group, single-center, open-label, exploratory, prospective randomized clinical trial designed to evaluate the safety and feasibility of performing burr hole craniotomy for the evacuation of cSDH under GA compared with LA. The trial was conducted at a tertiary academic medical center in Germany. Ethics approval was obtained from the institutional review board (EA4/100/23). The study protocol is available at the German Clinical Trials Register (DRKS00034040), and the statistical analysis report is provided in Supplement 1. Between October 2023 and November 2024, patients presenting with a confirmed diagnosis of cSDH were screened for eligibility. Patients were eligible if they were 18 years or older, provided written informed consent, and had an indication for hematoma evacuation. Surgical indication was defined by at least one of the following criteria: hematoma size exceeding skull thickness, ventricular compression, midline shift, neurological deficits, reduced level of consciousness, or hematoma progression. Exclusion criteria were preoperative delirium, contraindications to LA/GA, delay of more than 24 hours between diagnosis and surgery, presence of a ventricular shunt, need for bilateral hematoma evacuation, previous participation in the trial, and inability to provide informed consent. The sample size was calculated based on feasibility, estimating a recruitment rate of 40–50% of 120–150 patients operated annually. A feasibility limit was set at an actual recruitment rate above 21%. Patients meeting all inclusion criteria were randomly assigned in a 1:1 ratio to undergo surgery under either GA (propofol and remifentanil) or LA (bupivacaine and lidocaine). Importantly, LA was performed without the use of additional sedatives. An anesthesiologist was present on standby for all LA procedures to monitor patient discomfort. Both groups underwent identical treatment, consisting of a single parietal burr hole craniotomy, irrigation with normal saline, and subdural placement of an active drain for 48 hours. Given the recommendation against routine postoperative cranial imaging after cSDH evacuation, examinations were performed only in cases of clinical deterioration or persistent neurological deficits [9]. Postoperative radiological findings were further classified as recurrent cSDH requiring reoperation or as radiological residual cSDH, defined as residual hematoma on imaging without indication for surgical intervention. Randomization was performed using a computer-generated allocation sequence via a secure web-based system with concealed allocation. Data were recorded in electronic case report forms using REDCap (Vanderbilt University, Nashville, USA).

The primary endpoints were the rates of postoperative delirium and complications per patient until discharge, and the recruitment rate. Delirium was assessed by physicians every 8 hours using the Confusion Assessment Method (CAM), a widely established and validated tool that captures four core features of delirium: acute onset or fluctuating course, inattention, disorganized thinking, and altered level of consciousness [10]. All serial CAM assessments were aggregated to derive a binary outcome per patient (any delirium vs. none) for analysis. All postoperative complications were recorded without predefined restrictions, graded according to the Clavien-Dindo classification system (highest grade per patient) [11] and quantified using the Comprehensive Complication Index (CCI) [12].

Secondary endpoints included clinical parameters such as the Glasgow Coma Scale (GCS) [13], the modified Rankin Scale (mRS) [14, 15], and the Markwalder cSDH grading [16], assessed at admission, discharge, and 30 days postoperatively. Additional measures included preoperative laboratory values, procedural times, and length of hospital stay. Hematoma volume was determined by semi-automated segmentation using iPlanNet Cranial 3.0 software (Brainlab AG, Munich, Germany).

Statistical analyses were performed using the Brunner-Munzel test for ordinal and continuous variables, and the chi-squared test for nominal variables. Given the exploratory design of this trial, corrections for multiple comparisons were not performed. Outcome analyses were based on a per-protocol approach. All analyses were conducted using R software (R Foundation for Statistical Computing, Vienna, Austria), and reporting followed the CONSORT guidelines.

## Results

Of 167 patients screened for eligibility, 50 were enrolled and underwent surgery according to randomization (Supplementary Fig. 1A). Baseline characteristics were comparable between groups (Table 1). The primary endpoint analysis showed a significantly lower rate of postoperative delirium in the LA group (4%) compared with the GA group (32%; *p* = 0.03), corresponding to an odds ratio (OR) of 0.09 (95% confidence interval [CI], 0.01–0.79) and a number needed to treat (NNT) of 4 (Table 2, Supplementary Fig. 1B, Supplement 1). The overall complication rate was 8% in the LA group and 32% in the GA group (OR 0.18; 95% CI, 0.03–0.96; *p* = 0.08; NNT = 5). The severity of complications was comparable between groups.

**Table 1:** Baseline demographic and clinical characteristics of study participants at admission

		Overall (*n* = 50)	GA (*n* = 25)	LA (*n* = 25)	WMW Effect
Age, mean (SD), years		77.7 (9.8)	77.3 (10.4)	78.1 (9.3)	0.5 (0.3–0.6)
Sex, n (%), female		12 (24)	5 (20)	7 (28)	
Sex, n (%), male		38 (76)	20 (80)	18 (72)	
Bilateral cSDH, n (%)		2 (4)	1 (4)	1 (4)	
Laterality of cSDH, n (%), left		21 (42)	12 (48)	9 (36)	
Hematoma volume, mean (SD), cm^3^		110.4 (39.8)	105.1 (43.5)	115.7 (35.7)	0.4 (0.3–0.6)
Mixed-density hematoma, n (%)		16 (32)	8 (32)	8 (32)	
Comorbidities, n (%)		37 (74)	19 (76)	18 (72)	
History of head trauma, n (%)		32 (64)	13 (52)	19 (76)	
Antiplatelet agents, n (%)		14 (28)	7 (28)	7 (28)	
Anticoagulant agents, n (%)		14 (28)	6 (24)	8 (32)	
Laboratory values, mean (SD)	Quick, %	101.7 (16.8)	100.7 (19.0)	102.6 (14.6)	0.5 (0.3–0.7)
	INR	1 (0.2)	1 (0.3)	1 (0.1)	0.5 (0.3–0.7)
	aPTT, s	30.8 (11.9)	32.4 (16.4)	29.1 (3.7)	0.5 (0.3–0.7)
	Platelets, /nl	226.8 (54.9)	214.8 (49.0)	238.9 (58.7)	0.4 (0.2–0.6)
	RBC, /pl	4.3 (0.6)	4.2 (0.5)	4.5 (0.6)	0.3 (0.2–0.5)
	Hemoglobin, g/dl	13.3 (1.5)	13.0 (1.3)	13.7 (1.6)	0.4 (0.2–0.5)
	WBC, /nl	8.0 (2.2)	7.5 (2.0)	8.4 (2.2)	0.4 (0.3–0.6)
GCS, n (%)	12	1 (2)	0 (0)	1 (4)	0.4 (0.3–0.5)
	14	14 (28)	10 (40)	4 (16)	
	15	35 (70)	15 (60)	20 (80)	
mRS, n (%)	0	1 (2)	1 (4)	0 (0)	0.5 (0.4–0.7)
	1	10 (20)	3 (12)	7 (28)	
	2	20 (40)	10 (40)	10 (40)	
	3	12 (24)	9 (36)	3 (12)	
	4	7 (14)	2 (8)	5 (20)	
Markwalder grading, (%)	0	2 (4)	2 (8)	0 (0)	0.4 (0.3–0.6)
	1	22 (44)	11 (44)	11 (44)	
	2	26 (52)	12 (48)	14 (56)	

Continuous variables are presented as mean (± standard deviation), and categorical variables as absolute (relative) frequencies. Statistical significance was assessed using the Brunner-Munzel test for ordinal and continuous variables, and the chi-squared test for nominal variables. Effects are presented with 95% confidence intervals (CI) in parentheses. aPTT=activated partial thromboplastin time; GA=general anesthesia; GCS=Glasgow Coma Scale; INR=international normalized ratio; LA=local anesthesia; mRS=modified Rankin Scale; RBC = red blood cells; SD=standard deviation; WBC=white blood cells; WMW=Wilcoxon-Mann-Whitney

**Table 2:** Postoperative and 30-day follow-up outcomes

		Overall (*n* = 50)	GA (*n* = 25)	LA (*n* = 25)	WMW Effect	*p*-value
**Hospital stay**						
Delirium rate until discharge^a^, n (%)		9 (18)	8 (32)	1 (4)		**0.03**
Operating suite time^b^, mean (SD), min		168.3 (59.3)	196.0 (52.1)	139.3 (53.0)	0.8 (0.6–0.9)	**0.002**
Skin-to-skin time, mean (SD), min		40.4 (16.1)	40.6 (16.6)	40.2 (16.0)	0.5 (0.4–0.7)	0.8
Hospital stay, mean (SD), days		5.0 (4.7)	4.6 (3.6)	5.3 (5.7)	0.5 (0.3–0.7)	0.96
Complication rate until discharge^a^, n (%)		10 (20)	8 (32)	2 (8)		0.08
CCI, mean (SD)		41.0 (27.3)	36.5 (27.9)	59.3 (20.4)	0.2 (0-0.6)	0.12
Clavien-Dindo classification^c^, n (%)	II	7 (14)	7 (28)	0 (0)	0.1 (0-0.6)	0.09
	IIIb	2 (4)	0 (0)	2 (8)		
	V	1 (2)	1 (4)	0 (0)		
GCS at discharge, n (%)	14	10 (20)	7 (28)	3 (12)	0.4 (0.3–0.5)	0.15
	15	39 (78)	17 (68)	22 (88)		
	NA	1 (2)	1 (4)	0 (0)		
mRS at discharge, n (%)	0	21 (42)	8 (32)	13 (52)	0.6 (0.5–0.7)	0.16
	1	14 (28)	8 (32)	6 (24)		
	2	3 (6)	2 (8)	1 (4)		
	3	10 (20)	5 (20)	5 (20)		
	4	1 (2)	1 (4)	0 (0)		
	6	1 (2)	1 (4)	0 (0)		
Markwalder grading at discharge, n (%)	0	24 (48)	11 (44)	13 (52)	0.5 (0.4–0.7)	0.67
	1	25 (50)	13 (52)	12 (48)		
	NA	1 (2)	1 (4)	0 (0)		
**30-day follow-up**						
Loss to follow-up^d^, n (%)		2 (4)	0 (0)	2 (4)		
Complication rate from discharge until 30-day follow-up^a^, n (%)		12 (25.5)	7 (29.2)	5 (21.7)		0.8
GCS at 30-day follow-up, n (%)	12	1 (2)	1 (4)	0 (0)	0.4 (0.4–0.5)	0.17
	14	4 (8)	3 (12)	1 (4)		
	15	41 (82)	20 (80)	21 (84)		
	NA	4 (8)	1 (4)	3 (12)		
mRS at 30-day follow-up, n (%)	0	23 (46)	9 (36)	14 (56)	0.6 (0.4–0.7)	0.18
	1	10 (20)	7 (28)	3 (12)		
	2	1 (2)	1 (4)	0 (0)		
	3	11 (22)	6 (24)	5 (20)		
	4	1 (2)	1 (4)	0 (0)		
	6	2 (4)	1 (4)	1 (4)		
	NA	2 (4)	0 (0)	2 (8)		
Markwalder grading at 30-day follow-up, n (%)	0	28 (56)	12 (48)	16 (64)	0.6 (0.5–0.7)	0.15
	1	16 (32)	11 (44)	5 (20)		
	2	2 (4)	1 (4)	1 (4)		
	NA	4 (8)	1 (4)	3 (12)		

Continuous variables are presented as mean (± standard deviation), and categorical variables as absolute (relative) frequencies. Statistical significance was assessed using the Brunner-Munzel test for ordinal and continuous variables, and the chi-squared test for nominal variables. Effects are presented with 95% confidence intervals (CI) in parentheses. Bold values indicate statistical significance (*p* < 0.05). CCI=Comprehensive Complication Index; GA=general anesthesia; GCS=Glasgow Coma Scale; LA=local anesthesia; mRS=modified Rankin Scale; NA= not applicable; SD=standard deviation; WMW=Wilcoxon-Mann-Whitney

^a^ Evaluated as a binary outcome per patient

^b^ Operating suite time refers to the entire perioperative workflow: induction of GA or LA, patient positioning, surgical procedure, anesthesia management, and patient transfer

^c^ Highest grade per patient

^d^ Two patients in the LA group could not be contacted at follow-up and were excluded from the analysis. One patient in each group died: one in the GA group during the initial hospital stay and one in the LA group within the 30-day follow-up period after discharge

The total perioperative time spent in the operating suite, including induction of GA or LA, patient positioning, surgical procedure, anesthesia management, and patient transfer, was shorter in the LA group (139.3 ± 53.0 vs. 196.0 ± 52.1 min, *p* = 0.002). In contrast, skin-to-skin time and lengths of hospital stay were comparable between groups (Table 2, Supplementary Fig. 1C and 1D). There were no reports of perioperative patient discomfort requiring intervention or crossover in the LA group.

At 30-day follow-up, one patient in the GA group died during the initial hospital stay, and one patient in the LA group died after discharge. Additionally, two patients in the LA group were lost to follow-up. Complication rates and severity did not differ between LA (21.7%) and GA (29.2%) (OR 0.67; 95% CI, 0.18–2.52; *p* = 0.8). Detailed complications by group and per patient are provided in Supplement 1 (Tables 4a and 8a). Functional outcomes (GCS, mRS, Markwalder grading) were comparable between groups.

## Discussion

In this safety and feasibility trial, the rate of postoperative delirium was significantly lower in patients undergoing burr hole craniotomy for cSDH under LA compared with GA. Importantly, potential mediating factors such as hematoma volume, use of anticoagulant and antiplatelet medications, and laboratory values were comparable between groups. No safety concerns were identified, and complication rates and severity as well as functional outcomes were similar between groups. No crossover occurred, and procedural times and lengths of hospital stay were not prolonged in the LA group. These findings suggest that LA may be a feasible and well-tolerated alternative in burr hole craniotomy for cSDH, without raising safety concerns.

Available prospective studies on LA for cSDH involved the additional administration of systemic medications, such as sedatives or antipsychotics, which may influence the incidence of postoperative delirium, although this outcome was not systematically assessed in these studies [17, 18]. In contrast, our study aimed to minimize pharmacological co-interventions in order to better isolate the effect of the anesthetic modality on postoperative delirium and complications. To our knowledge, the present study is the first prospective clinical trial to systematically assess the effect of LA on the risk of postoperative delirium in cranial surgery, suggesting a potential protective effect for the vulnerable population of patients with cSDH and warranting further evaluation in neurosurgical practice and beyond.

The ABC-SDH trial was an exploratory single-center trial with a limited sample size, designed to assess safety and generate data to support the planning of a future phase III trial. The present study was conducted using an open-label design, as the nature of the intervention precluded blinding of patients or operating surgeons. To reduce assessor-related bias, CAM assessments were conducted by physicians independent of the operating team whenever feasible and were performed every 8 hours until discharge, thereby frequently involving different physicians. Nevertheless, as treatment allocation was likely apparent to assessors, the open-label design may have introduced detection bias in the clinical assessment of delirium and is therefore acknowledged as a limitation. Furthermore, the lack of objective biomarkers for delirium limited the objectivity of primary outcome assessment and may have contributed to residual bias [19]. To increase sensitivity in this exploratory study, delirium was classified as a binary outcome of any versus no delirium. This approach may obscure clinically relevant differences in delirium burden. Future phase III trials should therefore incorporate more granular, continuous delirium-related metrics, as well as standardized patient-reported measures of perioperative comfort, to better characterize delirium and its determinants.

Given the current recommendation, postoperative CT was performed only in patients with clinical deterioration or persistent neurological deficits [9]. As a result, postoperative pneumocephalus as well as the incidence and volume of recurrent cSDH could not be systematically assessed, although these factors may influence the incidence of postoperative delirium.

Owing to the lack of prior comparative data, a formal sample size calculation was not feasible. Of the 167 patients initially screened, 117 were excluded, primarily due to disorientation or impaired vigilance precluding informed consent. Despite the high exclusion rate, a recruitment rate of 0.3 (95% CI, 0.24–0.37) was achieved, notably exceeding the predefined feasibility threshold. These findings support the overall feasibility of the study design.

## Conclusion

In the ABC-SDH safety and feasibility trial, burr hole craniotomy for cSDH under LA was associated with a significantly lower rate of postoperative delirium compared with GA, without compromising surgical safety, procedural times, or functional outcomes. These findings highlight the potential advantages, feasibility, and safety of LA in a delirium-prone population. Larger multicenter trials are warranted to confirm these findings and to determine whether the observed reduction in postoperative delirium translates into improved clinical outcomes.

## Supplementary Information

Below is the link to the electronic supplementary material.

Supplementary Material 1.

Supplementary Material 2.

## Data Availability

The data that support the findings of this study are available from the corresponding author upon reasonable request. Access is restricted to protect patient confidentiality. Extended descriptive statistics are available through the attached statistical analysis report (Supplement 1). Analysis code is available at https://github.com/spruenke/ABC_SDH.

## Abbreviations

ABC: SDH-Awake Burr Hole Craniotomy for Chronic Subdural Hematoma
aPTT: Activated Partial Thromboplastin Time
CAM: Confusion Assessment Method
CCI: Comprehensive Complication Index
CI: Confidence Interval
cSDH: Chronic Subdural Hematoma
GA: General Anesthesia
GCS: Glasgow Coma Scale
INR: International Normalized Ratio
LA: Local Anesthesia
mRS: Modified Rankin Scale
OR: Odds Ratio
RBC: Red Blood Cells
SD: Standard Deviation
WBC: White Blood Cells
WMW: Wilcoxon-Mann-Whitney

## Declarations

### Trial registry

The full trial protocol is available at the German Clinical Trials Register (DRKS00034040), https://drks.de/search/en/trial/DRKS00034040/entails. Date of registration: 2024-04-15. The original study protocol, finalized before recruitment commenced, is provided as a supplementary file. All study planning, predefined endpoints, and statistical analyses were finalized prior to patient enrolment and conducted strictly according to the approved protocol.
