# Supplementary material for: Awake burr hole craniotomy for chronic subdural hematoma: a phase 2 randomized controlled trial
Source: Crit Care. 2026 Mar 9;30:120. doi: 10.1186/s13054-026-05913-1 (PMC12997716; doi:10.1186/s13054-026-05913-1)
Supplement: Supplementary file 1 — Supplementary Material 1. [file 13054_2026_5913_MOESM1_ESM.docx]

# Awake Burr Hole Craniotomy for Chronic Subdural Hematoma: A Phase 2 Randomized Controlled Trial

# Statistical Analysis Report

**Sample size calculation**

The sample size was determined with a focus on feasibility. Based on institutional data indicating that approximately 120 to 150 patients undergo surgery for chronic subdural hematoma (cSDH) each year, and assuming a 12-month recruitment period, a recruitment rate of 40% to 50% was estimated, yielding an expected sample size of approximately 50 patients. The primary planning parameter was the recruitment rate per group. For a recruitment rate of 40% and 25 patients per group, the 95% confidence interval (CI) ranges from 21% to 61%. If the actual recruitment rate does not fall below 21%, recruitment is considered feasible, and the data may serve as a basis for the planning of larger trials.

**Trial details**

No interim analyses were conducted, and no a priori stopping guidelines were set. The ABC-SDH trial was conducted as an open-label study, and patients, providers, and data analysts were aware of group allocation. There was no patient or public involvement in the design, conduct, or dissemination of the study. Trial registration: German Clinical Trials Register; ID: DRKS00034040; <https://drks.de/search/en/trial/DRKS00034040/entails>. Date of registration: April 15, 2024.

**Randomization**

Patients were randomly assigned in a 1:1 ratio using simple randomization without restrictions, blocking, or stratification, corresponding to a multiset permutation. The random allocation sequence was generated by the trial statisticians and implemented via the REDCap randomization module. Allocation was fully concealed within the REDCap system. Patients were assigned in the order of enrollment, based on the next entry in the pre-generated allocation list. Only the trial statisticians had access to the full allocation sequence. The enrolling investigators were blinded to upcoming allocations and had no access to the randomization sequence prior to assignment.

## Analysis set

A total of 50 unique patients were identified from the database.

Consequently, the analysis set comprised 50 unique patients for the period from baseline to discharge and 50 unique patients for the 30-day follow-up. For each variable, we report the number of non-missing observations in parentheses.

These patients correspond to the following recruitment rates (Wilson-Score CI in parentheses):

- Consent rate: Of 167 screened patients, after exclusion of further 88 patients, 28 of the remaining 79 declined to participate: 0.65 (0.54-0.74)
- Recruitment rate: Of 167 screened patients, 117 were excluded, and 50 remained for randomization: 0.3 (0.24-0.37)

For both CI (and consequently the point estimators), the lower bound lies above the 21% boundary that was required during the sample size planning to deem the trial feasible in terms of recruitment.

## Statistical analysis

This study employs a mainly descriptive approach to analyze the data. Summary statistics are presented for all variables of interest:

- For continuous variables, both mean and standard deviation are reported alongside Tukey’s five-number summary (minimum, 25th percentile, median, 75th percentile, maximum).
- For categorical variables, absolute frequencies (n) as well as relative frequencies (%) with respect to treatment group size are reported.
- To compare two groups of continuous or at least ordinally scaled variables regarding their stochastic dominance, the Brunner-Munzel Test is employed. The underlying statistical effect (Wilcoxon-Mann-Whitney Effect; WMW Effect) will also be reported and describes whether one group tends to have larger or smaller values than the other. In our case, this represents the probability that the local anesthesia group (LA) has smaller values than the general anesthesia group (GA), i.e., $P\left( X_{LA}< X_{GA} \right)+0.5\cdot P(X_{LA}=X_{GA})$. Thus, a WMW Effect smaller than 0.5 implies that the LA group stochastically tends to have larger values than the GA group. This procedure offers greater robustness than parametric tests, such as the t-test.
- To compare the distributions of categorical-nominal variables, Chi-Square Tests are employed.
- If not stated otherwise, numbers have been rounded to the first decimal. *P*-values have been formatted according to the formatting style of the JAMA Network.
- For CI, we generally employ a confidence level of $1 -\alpha= 0.95$. For means, a t-distribution has been used; for the WMW Effect, an asymptotic logit approximation was employed, and for proportions, a Wilson-Score interval based on the normal distribution has been used. 95% CI are reported in parentheses after the respective effect size.
- *P*-values should be interpreted cautiously and in a descriptive manner. Especially for the employed chi-square-tests, asymptotic requirements are not met.
- Due to the exploratory and descriptive nature of the study, no correction for multiple testing was employed.
- Missing values are reported. Missing values are not imputed. For each categorical variable, we report relative frequencies for all available data and for non-missing data.

### Descriptive Statistics

#### Characteristics at Baseline

| Variable | Metric | LA | GA | Total | WMW Effect |
| --- | --- | --- | --- | --- | --- |
| Age (years) (n LA: 25; n GA: 25) | Mean | 78.1 (74.3-81.9) | 77.3 (73-81.6) | 77.7 (74.9-80.5) | 0.5 (0.3-0.6) |
|  | SD | 9.3 | 10.4 | 9.8 |  |
|  | Min | 50 | 57 | 50 |  |
|  | 25% Qt. | 73 | 71 | 72 |  |
|  | Median | 81 | 80 | 80 |  |
|  | 75% Qt. | 85 | 86 | 85.8 |  |
|  | Max | 89 | 92 | 92 |  |
| Hematoma volume (cm^3^) (n LA: 25; n GA: 25) | Mean | 115.7 (101-130.4) | 105.1 (87.1-123.1) | 110.4 (99.1-121.7) | 0.4 (0.3-0.6) |
|  | SD | 35.7 | 43.5 | 39.8 |  |
|  | Min | 58 | 33 | 33 |  |
|  | 25% Qt. | 83 | 76 | 79.2 |  |
|  | Median | 116 | 91 | 104.5 |  |
|  | 75% Qt. | 145 | 130 | 138.5 |  |
|  | Max | 173 | 212 | 212 |  |
| Quick (%) (n LA: 25; n GA: 25) | Mean | 102.6 (96.6-108.6) | 100.7 (92.9-108.5) | 101.7 (96.9-106.5) | 0.5 (0.3-0.7) |
|  | SD | 14.6 | 19 | 16.8 |  |
|  | Min | 69 | 28 | 28 |  |
|  | 25% Qt. | 93 | 91 | 92.2 |  |
|  | Median | 103 | 104 | 103 |  |
|  | 75% Qt. | 114 | 114 | 114 |  |
|  | Max | 129 | 120 | 129 |  |
| INR (n LA: 25; n GA: 25) | Mean | 1 (1-1) | 1 (0.9-1.1) | 1 (0.9-1.1) | 0.5 (0.3-0.7) |
|  | SD | 0.1 | 0.3 | 0.2 |  |
|  | Min | 0.9 | 0.9 | 0.9 |  |
|  | 25% Qt. | 0.9 | 0.9 | 0.9 |  |
|  | Median | 1 | 1 | 1 |  |
|  | 75% Qt. | 1 | 1 | 1 |  |
|  | Max | 1.2 | 2.3 | 2.3 |  |
| aPTT (s) (n LA: 25; n GA: 25) | Mean | 29.1 (27.6-30.6) | 32.4 (25.6-39.2) | 30.8 (27.4-34.2) | 0.5 (0.3-0.7) |
|  | SD | 3.7 | 16.4 | 11.9 |  |
|  | Min | 21 | 23.6 | 21 |  |
|  | 25% Qt. | 26.8 | 26.1 | 26.2 |  |
|  | Median | 29.3 | 29.4 | 29.4 |  |
|  | 75% Qt. | 31.9 | 31.6 | 31.8 |  |
|  | Max | 36.9 | 109 | 109 |  |
| Platelets (/nl) (n LA: 25; n GA: 25) | Mean | 238.9 (214.7-263.1) | 214.8 (194.6-235) | 226.8 (211.2-242.4) | 0.4 (0.2-0.6) |
|  | SD | 58.7 | 49 | 54.9 |  |
|  | Min | 141 | 121 | 121 |  |
|  | 25% Qt. | 185 | 175 | 183.5 |  |
|  | Median | 241 | 210 | 236 |  |
|  | 75% Qt. | 266 | 250 | 250.8 |  |
|  | Max | 401 | 329 | 401 |  |
| RBC (/pl) (n LA: 25; n GA: 25) | Mean | 4.5 (4.3-4.7) | 4.2 (4-4.4) | 4.3 (4.1-4.5) | 0.3 (0.2-0.5) |
|  | SD | 0.6 | 0.5 | 0.6 |  |
|  | Min | 2.9 | 3 | 2.9 |  |
|  | 25% Qt. | 4.3 | 3.9 | 4 |  |
|  | Median | 4.5 | 4.1 | 4.4 |  |
|  | 75% Qt. | 4.9 | 4.6 | 4.7 |  |
|  | Max | 5.3 | 4.8 | 5.3 |  |
| Hemoglobin (g/dl) (n LA: 25; n GA: 25) | Mean | 13.7 (13-14.4) | 13 (12.5-13.5) | 13.3 (12.9-13.7) | 0.4 (0.2-0.5) |
|  | SD | 1.6 | 1.3 | 1.5 |  |
|  | Min | 9.6 | 10.1 | 9.6 |  |
|  | 25% Qt. | 12.9 | 12.5 | 12.6 |  |
|  | Median | 13.7 | 13.2 | 13.3 |  |
|  | 75% Qt. | 15.2 | 14.3 | 14.4 |  |
|  | Max | 15.8 | 14.8 | 15.8 |  |
| WBC (/nl) (n LA: 25; n GA: 25) | Mean | 8.4 (7.5-9.3) | 7.5 (6.7-8.3) | 8 (7.4-8.6) | 0.4 (0.3-0.6) |
|  | SD | 2.2 | 2 | 2.2 |  |
|  | Min | 5.9 | 3.5 | 3.5 |  |
|  | 25% Qt. | 7 | 6.2 | 6.5 |  |
|  | Median | 7.5 | 8 | 7.6 |  |
|  | 75% Qt. | 9.6 | 8.7 | 8.8 |  |
|  | Max | 14.8 | 11.6 | 14.8 |  |

**Table 1.** Continuous variables assessed at baseline. In each group, data were available from 25 (100%) patients. Effects are presented with 95% CI in parentheses. Abbreviations: aPTT = activated partial thromboplastin time, GA = general anesthesia, INR = international normalized ratio, LA = local anesthesia, RBC = red blood cells, SD = standard deviation, WBC = white blood cells, WMW = Wilcoxon-Mann-Whitney.

| Variable | Value | n  (LA) | %  (LA) | n  (GA) | %  (GA) | n (Total) | % (Total) |
| --- | --- | --- | --- | --- | --- | --- | --- |
| Sex (n LA: 25; n GA: 25) | Female | 7 | 28 | 5 | 20 | 12 | 24 |
|  | Male | 18 | 72 | 20 | 80 | 38 | 76 |
| Laterality of cSDH (n LA: 25; n GA: 25) | bilateral | 1 | 4 | 1 | 4 | 2 | 4 |
|  | left | 9 | 36 | 12 | 48 | 21 | 42 |
|  | right | 15 | 60 | 12 | 48 | 27 | 54 |
| Preoperative delirium (CAM) (n LA: 25; n GA: 25) | No | 25 | 100 | 25 | 100 | 50 | 100 |
| Comorbidities (n LA: 25; n GA: 25) | No | 7 | 28 | 6 | 24 | 13 | 26 |
|  | Yes | 18 | 72 | 19 | 76 | 37 | 74 |
| History of head trauma (n LA: 25; n GA: 25) | No | 6 | 24 | 12 | 48 | 18 | 36 |
|  | Yes | 19 | 76 | 13 | 52 | 32 | 64 |
| Antiplatelet agents (n LA: 25; n GA: 25) | No | 18 | 72 | 18 | 72 | 36 | 72 |
|  | Yes | 7 | 28 | 7 | 28 | 14 | 28 |
| Anticoagulant agents (n LA: 25; n GA: 25) | No | 17 | 68 | 19 | 76 | 36 | 72 |
|  | Yes | 8 | 32 | 6 | 24 | 14 | 28 |
| Mixed-density hematoma (n LA: 25; n GA: 25) | No | 17 | 68 | 17 | 68 | 34 | 68 |
|  | Yes | 8 | 32 | 8 | 32 | 16 | 32 |

**Table 2.** Categorical variables assessed at baseline. In each group, data were available from 25 (100%) patients. Data is presented in absolute (n) and relative (%) frequencies. Abbreviations: CAM = confusion assessment method, GA = general anesthesia, LA = local anesthesia.

| Variable | Value | n  (LA) | %  (LA) | n  (GA) | %  (GA) | n  (Total) | %  (Total) | WMW Effect |
| --- | --- | --- | --- | --- | --- | --- | --- | --- |
| GCS (n LA: 25; n GA: 25) | 12 | 1 | 4 | 0 | 0 | 1 | 2 | 0.4 (0.3-0.5) |
|  | 14 | 4 | 16 | 10 | 40 | 14 | 28 |  |
|  | 15 | 20 | 80 | 15 | 60 | 35 | 70 |  |
| mRS (n LA: 25; n GA: 25) | 0 | 0 | 0 | 1 | 4 | 1 | 2 | 0.5 (0.4-0.7) |
|  | 1 | 7 | 28 | 3 | 12 | 10 | 20 |  |
|  | 2 | 10 | 40 | 10 | 40 | 20 | 40 |  |
|  | 3 | 3 | 12 | 9 | 36 | 12 | 24 |  |
|  | 4 | 5 | 20 | 2 | 8 | 7 | 14 |  |
| Markwalder grading (n LA: 25; n GA: 25) | 0 | 0 | 0 | 2 | 8 | 2 | 4 | 0.4 (0.3-0.6) |
|  | 1 | 11 | 44 | 11 | 44 | 22 | 44 |  |
|  | 2 | 14 | 56 | 12 | 48 | 26 | 52 |  |

#### Table 3. Clinical measures at baseline. In each group, data were available from 25 (100%) patients. Data is presented in absolute (n) and relative (%) frequencies. Effects are presented with 95% CI in parentheses. Abbreviations: GA = general anesthesia, GCS = Glasgow coma scale, LA = local anesthesia, mRS = modified Rankin scale, WMW = Wilcoxon-Mann-Whitney.

#### Characteristics at Discharge

For the analysis of discharge characteristics, the following aspects should be noted: A total of 10 patients experienced complications (GA: 8; LA: 2). Accordingly, patients without complications contribute missing values for certain variables that are not applicable in the absence of a complication (e.g., Clavien-Dindo classification). Similarly, one patient (GA: 1; LA: 0) died and thus produced missing values for non-applicable variables such as GCS. These represent the only sources of missing data. For improved granularity, values for complication and delirium rates were rounded to two decimal places rather than one.

**Primary endpoint: complication rates**

| Variable | Value | n  (LA) | %  (LA) | n  (GA) | %  (GA) | n  (Total) | %  (Total) | *p*-value |
| --- | --- | --- | --- | --- | --- | --- | --- | --- |
| Complications (n LA: 25; n GA: 25) | No | 23 | 92 | 17 | 68 | 40 | 80 | .08 |
|  | Yes | 2 | 8 | 8 | 32 | 10 | 20 |  |

**Table 4.** Complication rates until discharge. Data are presented as a binary outcome per patient; each patient was counted once regardless of the number or type of complications. In each group, data were available from 25 (100%) patients. Data is presented in absolute (n) and relative (%) frequencies. Abbreviations: GA = general anesthesia, LA = local anesthesia.

The proportion of complications (Wilson-Score CI in parentheses) is:

- LA Group: 0.08 (0.02-0.25)
- GA Group: 0.32 (0.17-0.52)
- Overall: 0.2 (0.11-0.33)

The odds ratio (OR) for experiencing complications until discharge in the LA group compared to the GA group, together with its asymptotic CI in parentheses is: 0.18 (0.03-0.96), i.e., the odds of having complications after surgery under LA are 0.18-fold. The Number Needed to Treat (NNT) is 5 (4.17).

**Detailed complications per patient**

| **GA Group** | | | | |
| --- | --- | --- | --- | --- |
| **Patient ID** | **Complications** | **Time to first delirium episode (hours)** | **Clavien-Dindo classification** | **Comprehensive Complication Index (CCI)** |
| 3 | Delirium | 40 | 2 | 20.9 |
| 14 | Delirium | 8 | 2 | 20.9 |
| 16 | Delirium, cortical subarachnoid hemorrhage, intracerebral hemorrhage | 8 | 2 | 36.2 |
| 23 | Delirium | 8 | 2 | 20.9 |
| 27 | Delirium, fatal outcome after a fall | 32 | 5 | 100.0 |
| 28 | Delirium, urinary tract infection, pneumonia, pleural effusions, epileptic seizure, radiological residual cSDH | 16 | 2 | 51.2 |
| 36 | Delirium | 8 | 2 | 20.9 |
| 37 | Delirium | 8 | 2 | 20.9 |
| **LA Group** | | | | |
| **Patient ID** | **Complications** | **Time to first delirium episode (hours)** | **Clavien-Dindo classification** | **Comprehensive Complication Index (CCI)** |
| 2 | Delirium, recurrent cSDH requiring four reoperations, pulmonary embolism | 8 | 3b | 73.7 |
| 35 | Recurrent cSDH requiring reoperation, epileptic seizure, pneumonia | n/a | 3b | 44.9 |

**Table 4a.** Detailed complications until discharge by group and per patient. For the Clavien-Dindo classification, the highest grade per patient is reported. Abbreviations: CCI = Comprehensive Complication Index, cSDH = chronic subdural hematoma, GA = general anesthesia, LA = local anesthesia

**Primary endpoint: delirium rates**

| Variable | Value | n  (LA) | %  (LA) | n  (GA) | %  (GA) | n  (Total) | %  (Total) | *p*-value |
| --- | --- | --- | --- | --- | --- | --- | --- | --- |
| Delirium (n LA: 25; n GA: 25) | No | 24 | 96 | 17 | 68 | 41 | 82 | .03 |
|  | Yes | 1 | 4 | 8 | 32 | 9 | 18 |  |

**Table 5.** Delirium rates until discharge. Data are presented as a binary outcome per patient; each patient was counted once regardless of delirium duration or recurrence. In each group, data were available from 25 (100%) patients. Data is presented in absolute (n) and relative (%) frequencies. Abbreviations: GA = general anesthesia, LA = local anesthesia.

The proportion of delirium (Wilson-Score CI in parentheses) is:

- LA Group: 0.04 (0.01-0.2)
- GA Group: 0.32 (0.17-0.52)
- Overall: 0.18 (0.1-0.31)

The OR for experiencing delirium until discharge in the LA group compared to the GA group, together with its asymptotic CI in parentheses is: 0.09 (0.01-0.79), i.e., the odds of having delirium after surgery under LA are 0.09-fold. The Number Needed to Treat (NNT) is 4 (3.57).

**Secondary endpoints at Discharge**

| Variable | Metric | LA | GA | Total | WMW Effect | *p*-value |
| --- | --- | --- | --- | --- | --- | --- |
| Duration of surgical team assignment (min) (n LA: 25; n GA: 25) | Mean | 127.2 (113.4-141) | 159.3 (145.8-172.8) | 143.2 (132.8-153.6) | 0.8 (0.6-0.9) | .005 |
|  | SD | 33.4 | 32.6 | 36.5 |  |  |
|  | Min | 66 | 90 | 66 |  |  |
|  | 25% Qt. | 104 | 139 | 117 |  |  |
|  | Median | 120 | 163 | 139 |  |  |
|  | 75% Qt. | 141 | 170 | 165 |  |  |
|  | Max | 202 | 230 | 230 |  |  |
| Skin-to-skin time (min) (n LA: 25; n GA: 25) | Mean | 40.2 (33.6-46.8) | 40.6 (33.7-47.5) | 40.4 (35.8-45) | 0.5 (0.4-0.7) | .8 |
|  | SD | 16 | 16.6 | 16.1 |  |  |
|  | Min | 17 | 8 | 8 |  |  |
|  | 25% Qt. | 29 | 30 | 29.2 |  |  |
|  | Median | 37 | 39 | 38 |  |  |
|  | 75% Qt. | 50 | 49 | 49.8 |  |  |
|  | Max | 80 | 73 | 80 |  |  |
| Operating suite time* (min) (n LA: 23; n GA: 24) | Mean | 139.3 (116.4-162.2) | 196 (174-218) | 168.3 (150.9-185.7) | 0.8 (0.6-0.9) | .002 |
|  | SD | 53 | 52.1 | 59.3 |  |  |
|  | Min | 73 | 115 | 73 |  |  |
|  | 25% Qt. | 98.5 | 168.2 | 120 |  |  |
|  | Median | 122 | 188.5 | 171 |  |  |
|  | 75% Qt. | 182.5 | 215.5 | 205 |  |  |
|  | Max | 267 | 298 | 298 |  |  |
|  | NA | 2 | 1 | 3 |  |  |
| Hospital stay (days) (n LA: 25; n GA: 25) | Mean | 5.3 (2.9-7.7) | 4.6 (3.1-6.1) | 5 (3.7-6.3) | 0.5 (0.3-0.7) | .96 |
|  | SD | 5.7 | 3.6 | 4.7 |  |  |
|  | Min | 2 | 1 | 1 |  |  |
|  | 25% Qt. | 3 | 3 | 3 |  |  |
|  | Median | 3 | 3 | 3 |  |  |
|  | 75% Qt. | 6 | 4 | 5.8 |  |  |
|  | Max | 30 | 18 | 30 |  |  |
| Comprehensive Complication Index (CCI) (n LA: 2; n GA: 8) | Mean | 59.3 (-124-242.6) | 36.5 (13.2-59.8) | 41 (21.5-60.5) | 0.2 (0-0.6) | .12 |
|  | SD | 20.4 | 27.9 | 27.3 |  |  |
|  | Min | 44.9 | 20.9 | 20.9 |  |  |
|  | 25% Qt. | 52.1 | 20.9 | 20.9 |  |  |
|  | Median | 59.3 | 20.9 | 28.6 |  |  |
|  | 75% Qt. | 66.5 | 40 | 49.6 |  |  |
|  | Max | 73.7 | 100 | 100 |  |  |
|  | NA | 23 | 17 | 40 |  |  |

**Table 6.** Procedural times and Comprehensive Complication Index (CCI) at discharge. In groups where data is not available for all patients, the number of non-available data points is given as NA. Effects are presented with 95% CI in parentheses. Abbreviations: CCI = Comprehensive Complication Index, GA = general anesthesia, LA = local anesthesia, SD = standard deviation, WMW = Wilcoxon-Mann-Whitney.

*Operating suite time refers to the perioperative duration including induction of GA or preparation for LA, patient positioning, the surgical procedure itself, and postoperative measures such as anesthesia management and patient transfer.

| Variable | Value | n  (LA) | %  (LA) | % without NA  (LA) | n  (GA) | %  (GA) | % without NA  (GA) | n  (Total) | %  (Total) | % without NA (Total) | WMW Effect | *p*-value |
| --- | --- | --- | --- | --- | --- | --- | --- | --- | --- | --- | --- | --- |
| Clavien-Dindo classification (n LA: 2; n GA: 8) | Grade I | 0 | 0 | 0 | 0 | 0 | 0 | 0 | 0 | 0 | 0.1 (0-0.6) | .09 |
|  | Grade II | 0 | 0 | 0 | 7 | 28 | 87.5 | 7 | 14 | 70 |  |  |
|  | Grade IIIa | 0 | 0 | 0 | 0 | 0 | 0 | 0 | 0 | 0 |  |  |
|  | Grade IIIb | 2 | 8 | 100 | 0 | 0 | 0 | 2 | 4 | 20 |  |  |
|  | Grade IVa | 0 | 0 | 0 | 0 | 0 | 0 | 0 | 0 | 0 |  |  |
|  | Grade IVb | 0 | 0 | 0 | 0 | 0 | 0 | 0 | 0 | 0 |  |  |
|  | Grade V | 0 | 0 | 0 | 1 | 4 | 12.5 | 1 | 2 | 10 |  |  |
|  | NA | 23 | 92 |  | 17 | 68 |  | 40 | 80 |  |  |  |
| GCS (n LA: 25; n GA: 24) | 14 | 3 | 12 | 12 | 7 | 28 | 29.2 | 10 | 20 | 20.4 | 0.4 (0.3-0.5) | .15 |
|  | 15 | 22 | 88 | 88 | 17 | 68 | 70.8 | 39 | 78 | 79.6 |  |  |
|  | NA | 0 | 0 |  | 1 | 4 |  | 1 | 2 |  |  |  |
| mRS (n LA: 25; n GA: 25) | 0 | 13 | 52 | 52 | 8 | 32 | 32 | 21 | 42 | 42 | 0.6 (0.5-0.7) | .16 |
|  | 1 | 6 | 24 | 24 | 8 | 32 | 32 | 14 | 28 | 28 |  |  |
|  | 2 | 1 | 4 | 4 | 2 | 8 | 8 | 3 | 6 | 6 |  |  |
|  | 3 | 5 | 20 | 20 | 5 | 20 | 20 | 10 | 20 | 20 |  |  |
|  | 4 | 0 | 0 | 0 | 1 | 4 | 4 | 1 | 2 | 2 |  |  |
|  | 6 | 0 | 0 | 0 | 1 | 4 | 4 | 1 | 2 | 2 |  |  |
| Markwalder grading (n LA: 25; n GA: 24) | 0 | 13 | 52 | 52 | 11 | 44 | 45.8 | 24 | 48 | 49 | 0.5 (0.4-0.7) | .67 |
|  | 1 | 12 | 48 | 48 | 13 | 52 | 54.2 | 25 | 50 | 51 |  |  |
|  | NA | 0 | 0 |  | 1 | 4 |  | 1 | 2 |  |  |  |

**Table 7.** Secondary outcomes at discharge. In groups where data is not available for all patients, the number of non-available data points is given as NA. Effects are presented with 95% CI in parentheses. For the Clavien-Dindo classification, the highest grade per patient is reported. Abbreviations: GA = general anesthesia, GCS = Glasgow coma scale, LA = local anesthesia, mRS = modified Rankin scale, WMW = Wilcoxon-Mann-Whitney.

#### Characteristics at 30-Day Follow-Up

For the analysis of 30-day follow-up data, the following should be noted: two patients in the LA group could not be reached and were classified as lost to follow-up. These two patients were excluded from the follow-up analysis. One patient in the GA group died before discharge and one patient in the LA group died between discharge and follow-up. Complication rates and severity were analyzed from discharge until the 30-day follow-up. Further outcomes were analyzed from surgery until the 30-day follow-up. In analogy to the discharge analysis, patients without complications and patients who died contribute missing values for inapplicable variables (e.g., Clavien-Dindo classification or GCS, respectively). These were the only reasons for missing data after exclusion of the two lost-to-follow-up patients. For improved granularity, values for complication rates were rounded to two decimal places rather than one.

**Complication rates**

| Variable | Value | n  (LA) | %  (LA) | % without NA  (LA) | n  (GA) | %  (GA) | % without NA  (GA) | n  (Total) | %  (Total) | % without NA  (Total) | *p*-value |
| --- | --- | --- | --- | --- | --- | --- | --- | --- | --- | --- | --- |
| Complications (n LA: 23; n GA: 24) | No | 18 | 72 | 78.26 | 17 | 68 | 70.83 | 35 | 70 | 74.47 | .8 |
|  | Yes | 5 | 20 | 21.74 | 7 | 28 | 29.17 | 12 | 24 | 25.53 |  |
|  | NA | 2 | 8 |  | 1 | 4 |  | 3 | 6 |  |  |

**Table 8.** Complication rates from discharge until 30-day follow-up. Data are presented as a binary outcome per patient; each patient was counted once regardless of the number or type of complications. In groups where data is not available for all patients, the number of non-available data points is given as NA. Abbreviations: GA = general anesthesia, LA = local anesthesia.

The proportion of complications (Wilson-Score CI in parentheses) is:

- LA Group: 0.22 (0.1-0.42)
- GA Group: 0.29 (0.15-0.49)
- Overall: 0.26 (0.15-0.4)

The odds ratio (OR) for experiencing complications from discharge until the 30-day follow-up in the LA group compared to the GA group, together with its asymptotic CI in parentheses is: 0.67 (0.18-2.52), i.e., the odds of having complications after surgery under LA are 0.67-fold. The Number Needed to Treat (NNT) is 14 (13.46).

**Detailed complications per patient**

| **GA Group** | | | |
| --- | --- | --- | --- |
| **Patient ID** | **Complications** | **Clavien-Dindo classification** | **Comprehensive Complication Index (CCI)** |
| 1 | Radiological residual cSDH | 1 | 8.7 |
| 9 | Radiological residual cSDH, hygroma | 1 | 8.7 |
| 16 | Pneumonic sepsis, recurrent cSDH requiring reoperation | 4a | 54.2 |
| 21 | Radiological residual cSDH | 1 | 8.7 |
| 32 | Radiological residual cSDH | 1 | 8.7 |
| 37 | Recurrent cSDH requiring reoperation and embolization of the middle meningeal artery, TIA | 3b | 43.6 |
| 45 | Wound healing disorder | 1 | 8.7 |
| **LA Group** | | | |
| **Patient ID** | **Complications** | **Clavien-Dindo classification** | **Comprehensive Complication Index (CCI)** |
| 8 | Radiological residual cSDH | 1 | 8.7 |
| 26 | Delirium, pulmonary embolism requiring thrombectomy, radiological residual cSDH | 3b | 40.6 |
| 34 | Left-sided upper extremity deep vein thrombosis following pacemaker implantation | 2 | 20.9 |
| 39 | Death due to cardiac arrest | 5 | 100.0 |
| 43 | Wound healing disorder | 1 | 8.7 |

**Table 8a.** Detailed complications from discharge until 30-day follow-up by group and per patient. For the Clavien-Dindo classification, the highest grade per patient is reported. Abbreviations: CCI = Comprehensive Complication Index, cSDH = chronic subdural hematoma, GA = general anesthesia, LA = local anesthesia

##### Secondary endpoints at 30-day follow-up

| Variable | Metric | LA | GA | Total | WMW Effect | *p*-value |
| --- | --- | --- | --- | --- | --- | --- |
| Comprehensive Complication Index (CCI)  (n LA: 5; n GA: 7) | Mean | 35.8 (-11.6-83.2) | 20.2 (1.8-38.6) | 26.7 (8.6-44.8) | 0.4 (0.1-0.7) | .46 |
|  | SD | 38.2 | 19.9 | 28.5 |  |  |
|  | Min | 8.7 | 8.7 | 8.7 |  |  |
|  | 25% Qt. | 8.7 | 8.7 | 8.7 |  |  |
|  | Median | 20.9 | 8.7 | 8.7 |  |  |
|  | 75% Qt. | 40.6 | 26.1 | 41.4 |  |  |
|  | Max | 100 | 54.2 | 100 |  |  |
|  | NA | 20 | 18 | 38 |  |  |

**Table 9.** Comprehensive Complication Index (CCI) from discharge until 30-day follow-up. In groups where data is not available for all patients, the number of non-available data points is given as NA. Effects are presented with 95% CI in parentheses. Abbreviations: CCI = Comprehensive Complication Index, GA = general anesthesia, LA = local anesthesia, SD = standard deviation, WMW = Wilcoxon-Mann-Whitney.

| Variable | Value | n  (LA) | %  (LA) | % without NA  (LA) | n  (GA) | %  (GA) | % without NA  (GA) | n  (Total) | %  (Total) | % without NA  (Total) | WMW Effect | *p*-value |
| --- | --- | --- | --- | --- | --- | --- | --- | --- | --- | --- | --- | --- |
| GCS (n LA: 22; n GA: 24) | 12 | 0 | 0 | 0 | 1 | 4 | 4.2 | 1 | 2 | 2.2 | 0.4 (0.4-0.5) | .17 |
|  | 14 | 1 | 4 | 4.5 | 3 | 12 | 12.5 | 4 | 8 | 8.7 |  |  |
|  | 15 | 21 | 84 | 95.5 | 20 | 80 | 83.3 | 41 | 82 | 89.1 |  |  |
|  | NA | 3 | 12 |  | 1 | 4 |  | 4 | 8 |  |  |  |
| mRS (n LA: 23; n GA: 25) | 0 | 14 | 56 | 60.9 | 9 | 36 | 36 | 23 | 46 | 47.9 | 0.6 (0.4-0.7) | .18 |
|  | 1 | 3 | 12 | 13 | 7 | 28 | 28 | 10 | 20 | 20.8 |  |  |
|  | 2 | 0 | 0 | 0 | 1 | 4 | 4 | 1 | 2 | 2.1 |  |  |
|  | 3 | 5 | 20 | 21.7 | 6 | 24 | 24 | 11 | 22 | 22.9 |  |  |
|  | 4 | 0 | 0 | 0 | 1 | 4 | 4 | 1 | 2 | 2.1 |  |  |
|  | 6 | 1 | 4 | 4.3 | 1 | 4 | 4 | 2 | 4 | 4.2 |  |  |
|  | NA | 2 | 8 |  | 0 | 0 |  | 2 | 4 |  |  |  |
| Markwalder grading (n LA: 22; n GA: 24) | 0 | 16 | 64 | 72.7 | 12 | 48 | 50 | 28 | 56 | 60.9 | 0.6 (0.5-0.7) | .15 |
|  | 1 | 5 | 20 | 22.7 | 11 | 44 | 45.8 | 16 | 32 | 34.8 |  |  |
|  | 2 | 1 | 4 | 4.5 | 1 | 4 | 4.2 | 2 | 4 | 4.3 |  |  |
|  | NA | 3 | 12 |  | 1 | 4 |  | 4 | 8 |  |  |  |
| Clavien-Dindo classification (n LA: 5; n GA: 7) | Grade I | 2 | 8 | 40 | 5 | 20 | 71.4 | 7 | 14 | 58.3 | 0.4 (0.1-0.7) | .41 |
|  | Grade II | 1 | 4 | 20 | 0 | 0 | 0 | 1 | 2 | 8.3 |  |  |
|  | Grade IIIa | 0 | 0 | 0 | 0 | 0 | 0 | 0 | 0 | 0 |  |  |
|  | Grade IIIb | 1 | 4 | 20 | 1 | 4 | 14.3 | 2 | 4 | 16.7 |  |  |
|  | Grade IVa | 0 | 0 | 0 | 1 | 4 | 14.3 | 1 | 2 | 8.3 |  |  |
|  | Grade IVb | 0 | 0 | 0 | 0 | 0 | 0 | 0 | 0 | 0 |  |  |
|  | Grade V | 1 | 4 | 20 | 0 | 0 | 0 | 1 | 2 | 8.3 |  |  |
|  | NA | 20 | 80 |  | 18 | 72 |  | 38 | 76 |  |  |  |

**Table 10**. Secondary outcomes at 30-day follow-up**.** Data is presented in absolute (n) and relative (%) frequencies. Effects are presented with 95% CI in parentheses. For the Clavien-Dindo classification, the highest grade per patient is reported from discharge until 30-day follow-up. Abbreviations: GA = general anesthesia, GCS = Glasgow coma scale, LA = local anesthesia, mRS = modified Rankin scale, WMW = Wilcoxon-Mann-Whitney.

**Comparison over Time**

#### Flow of mRS over time

####
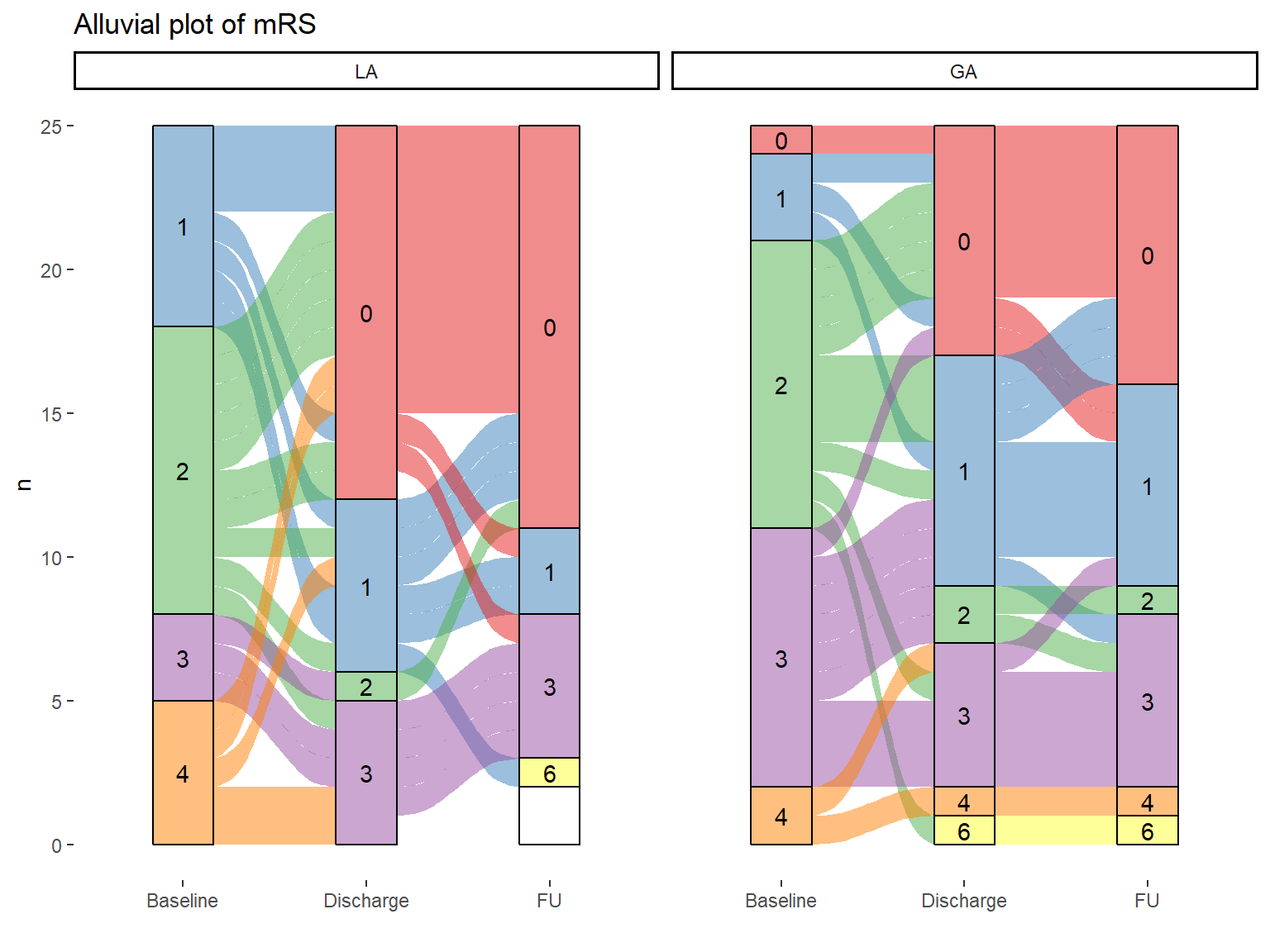


**Flow of Markwalder over time**

####
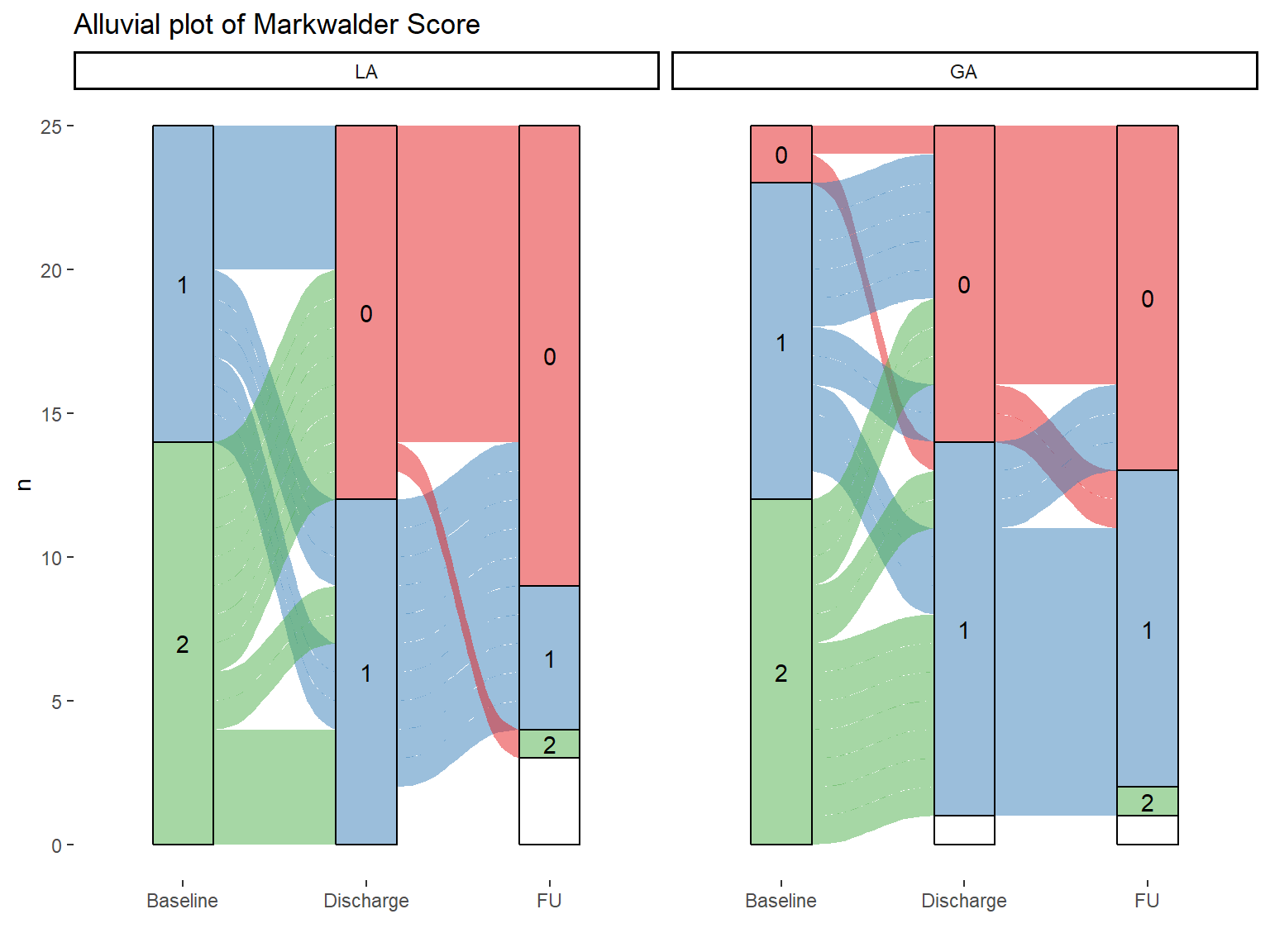


#### Software

R version 4.3.2 with packages

- nparcomp version 3.0
- dplyr version 1.1.4
- ggplot2 version 3.5.1
- ggalluvial version 0.12.5
- flextable version 0.9.7

Analysis code is available at: <https://github.com/spruenke/ABC_SDH>.
