# Supplementary material for: Awake burr hole craniotomy for chronic subdural hematoma: a phase 2 randomized controlled trial
Source: Crit Care. 2026 Mar 9;30:120. doi: 10.1186/s13054-026-05913-1 (PMC12997716; doi:10.1186/s13054-026-05913-1)
Supplement: Supplementary file 2 — Supplementary Material 2. [file 13054_2026_5913_MOESM2_ESM.docx]

**Supplementary Figure 1. Patient allocation, postoperative delirium rate, and procedural times. A** CONSORT flowchart depicting patient inclusion, allocation, and analysis. **B** Postoperative delirium rate following surgery under GA and LA. **C** Total time spent in the operating suite under GA and LA, encompassing the entire perioperative workflow: induction of GA or LA, patient positioning, surgical procedure, anesthesia management, and patient transfer. **D** Skin-to-skin time under GA and LA. Panels C and D show violin plots of time measurements. Individual dots represent patient-level data. Horizontal black bars indicate group means and vertical lines represent standard deviations. Violin width corresponds to estimated data density based on kernel density estimation. GA = general anesthesia; LA = local anesthesia.
